# Molecular detection of *Borrelia burgdorferi* (*sensu lato*) and *Rickettsia* spp. in hard ticks distributed in Tokachi District, eastern Hokkaido, Japan

**DOI:** 10.1016/j.crpvbd.2021.100059

**Published:** 2021-11-11

**Authors:** Kiyoshi Okado, Paul Franck Adjou Moumouni, Seung-Hun Lee, Thillaiampalam Sivakumar, Naoaki Yokoyama, Kozo Fujisaki, Hiroshi Suzuki, Xuenan Xuan, Rika Umemiya-Shirafuji

**Affiliations:** a National Research Center for Protozoan Diseases, Obihiro University of Agriculture and Veterinary Medicine, Hokkaido 080-8555, Japan; b National Agricultural and Food Research Organization, Kannondai 3-1-5, Ibaraki 305-0856, Japan

**Keywords:** *Ixodes ovatus*, *Ixodes persulcatus*, *Haemaphysalis japonica*, *Haemaphysalis megaspinosa*, *Borrelia*, *Rickettsia*

## Abstract

Ticks transmit various pathogens, including parasites, bacteria and viruses to humans and animals. To investigate the ticks and the potentially zoonotic pathogens that they may carry, questing ticks were collected in 2017 from 7 sites in Tokachi District, eastern Hokkaido, Japan. A total of 1563 ticks including adults (male and female), nymphs and larvae were collected. Four species of ticks were identified: *Ixodes ovatus*, *Ixodes persulcatus*, *Haemaphysalis japonica* and *Haemaphysalis megaspinosa*. Of the 1563 ticks, 1155 were used for DNA extraction. In total, 527 individual tick DNA samples prepared from adults (*n* = 484), nymphs (*n* = 41) and larvae (*n* = 2); and 67 pooled tick DNA samples prepared from larval stages (*n* = 628) were examined using PCR methods and sequencing to detect *Borrelia burgdorferi* (*sensu lato*) and *Rickettsia* spp. The phylogenetic analysis of *Borrelia* spp. *flaB* gene sequences showed the presence of the human pathogenic *B. burgdorferi* (*s.l.*) species (*Borrelia garinii*, *Borrelia bavariensis* and *Borrelia afzelii*) in *I*. *persulcatus*, whereas the non-pathogenic species *Borrelia japonica* was found only in *I. ovatus*. In *I*. *persulcatus*, *B. garinii* and/or its closely related species *B. bavariensis* was detected in both adults and nymphs at a prevalence of 21.9% whereas *B. afzelii* was found only in adults (1.8%). The prevalence of *B. japonica* in adult *I*. *ovatus* was 21.8%. *Rickettsia* species were identified through phylogenetic analysis based on *gltA*, 16S rRNA, *ompB* and *sca4* genes. Four genotypes were detected in the samples which were classified into three species. The prevalence of human pathogenic *Rickettsia helvetica* was 26.0% in *I. persulcatus* adults and nymphs, 55.6% in *I. persulcatus* larval pools, and 1.7% in *H. megaspinosa* larval pools. The prevalence of “*Candidatus* R. tarasevichiae” was 15.4% in *I. persulcatus* adults and nymphs and 33.3% in *I. persulcatus* larval pools. The prevalence of “*Candidatus* R. principis” in *H. megaspinosa* adults and nymphs was 11.1% whereas it was detected in 3.4% of the *H. megaspinosa* larval pools. These results indicate that most of the risks of Lyme borreliosis and spotted fever group rickettsiosis infection in eastern Hokkaido, Japan, are restricted to *I. persulcatus*.

## Highlights

• Four species of ticks were collected in Tokachi District, eastern Hokkaido, Japan.
• Tick DNA samples were subjected to PCR to detect *Borrelia* spp. and *Rickettsia* spp.
• *Borrelia garinii/Borrelia bavariensis* and *Borrelia afzelii* were detected in *Ixodes persulcatus.*
• *Rickettsia helvetica* and “*Candidatus* R. tarasevichiae” were detected in *I. persulcatus.*
• First molecular detection of “*Candidatus* R. principis” in larvae of *Haemaphysalis megaspinosa.*

## Graphical abstract

**Figure:**
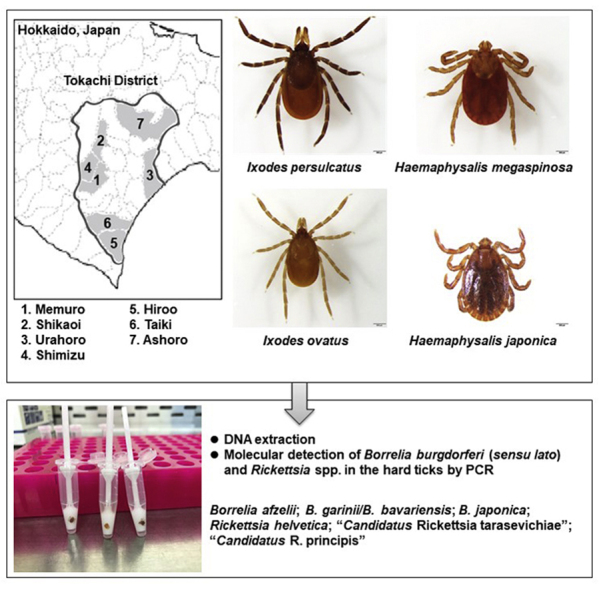

## 1 Introduction

In Japan located in East Asia, the dynamics of tick-borne pathogens is changing as the incidence of related human disease cases is increasing (Yamaji et al., 2018). Mapping of the distribution of ticks and the zoonotic pathogens they carry, in each locality of the country is one of the constant key efforts to assess the risk of the occurrence of infectious diseases. Tokachi District in eastern Hokkaido, Japan, is famous for its agriculture and dairy farming that utilize the areaʼs vast plains. Because dairy cows live in meadows shared with wild animals such as sika deer, they frequently suffer tick infestations while grazing, are at great risk of pathogenic infections (Ota et al., 2009; Shibata et al., 2018) and may serve as a source of infected ticks. Likewise, not only dairy farmers but also farmworkers and hunters are at high risk for tick bites which potentially transmit various tick-borne zoonotic diseases (Kubo et al., 1992; Chaligiannis et al., 2018). Moreover, outdoor nature-based recreational activities such as hiking, fishing and barbecuing are popular in the natural environment of Tokachi District surrounded by mountains and might also be associated with risks of tick bites.

Lyme borreliosis and rickettsioses are important tick-borne zoonotic diseases. Despite substantial efforts to improve surveillance and control of Lyme borreliosis, it remains prevalent in the northern hemisphere (Rizzoli et al., 2011). Most cases of Lyme borreliosis in Japan have been confirmed in Hokkaido and are caused by *Borrelia garinii* and *Borrelia afzelii* infections (IDSC, 2011). Previous research indicated that *Borrelia burgdorferi* (*sensu stricto*) has a greater inflammatory potential than *B. afzelii* or *B. garinii* (Strle et al., 2009), but *B. burgdorferi* (*s.s*.) has not yet been found in Japan (Masuzawa, 2004). As the geographical distribution of pathogens and the number of patients are ever changing in Japan, molecular epidemiological surveys for *Borrelia* spp. are required to assess the risk of infection in different geographical areas of the country. Tick-borne rickettsioses are globally distributed and one of the oldest known zoonoses (Parola et al., 2005). Multiple distinct tick-borne spotted fever group (SFG) rickettsioses have been recognized by the development of molecular methods to identify rickettsiae (Parola et al., 2005). From 1984 through 2005, 11 additional rickettsiae were described as causative agents of tick-borne rickettsioses (Raoult & Roux, 1997; Parola et al., 2005). Among them, seven were initially isolated from ticks, before their association with human disease was confirmed (Parola et al., 2005). At present, there are 25 pathogenic *Rickettsia* species in the world (Piotrowski & Rymaszewska, 2020). In Japan, notification of Japanese spotted fever (JSF) caused by *Rickettsia japonica* increases year by year, especially from 2007. In total, 2726 JSF patients were reported in 2007–2019 (IDSC, 2020), with the largest annual number of notifications (337 cases) being recorded in 2017. Several other SFG rickettsiae, namely *Rickettsia heilongjiangensis*, *Rickettsia helvetica*, *Rickettsia tamurae* and “*Candidatus* R. tarasevichiae”, have also been recognized as etiological agents of human diseases (IDSC, 2006; Ando et al., 2010; Jia et al., 2013; Liu et al., 2016). *Rickettsia asiatica*, has also been reported in Japan but its pathogenicity is currently unknown (Fujita et al., 2006). Molecular epidemiological surveys for *Borrelia* spp. and *Rickettsia* spp. in ticks provide useful data for the diagnosis and prevention of these tick-borne diseases (Eisen & Paddock, 2020).

In the present study, to investigate the zoonotic pathogens carried by ticks in Tokachi District, eastern Hokkaido, Japan, we collected questing ticks from seven sites of the district in 2017. The tick samples were identified to the species level, then selected specimens were submitted to DNA extraction, PCR and sequencing to detect *B. burgdorferi* (*s.l.*) and *Rickettsia* spp.

## 2 Materials and methods

### 2.1 Tick collection and identification

**Fig. 1:**
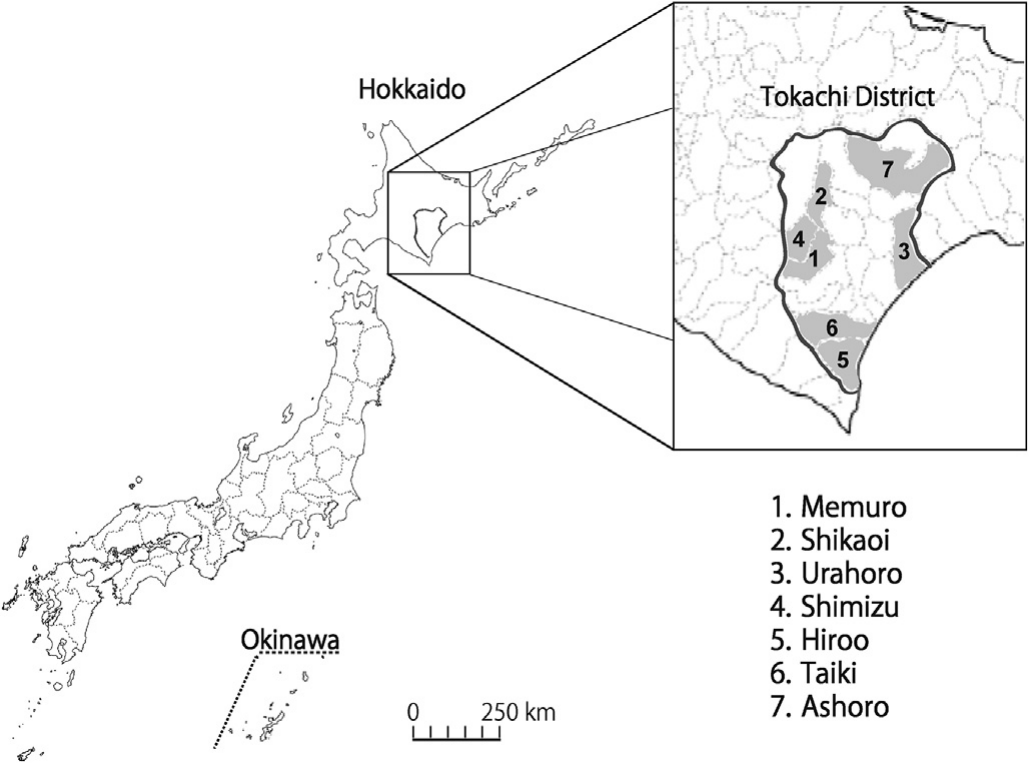
Tick collection sites in the Tokachi District (Hokkaido, Japan). We collected questing ticks throughout the district: two northern sites (Shikaoi and Ashoro), three central sites (Urahoro, Shimizu and Memuro) and two southern sites (Hiroo and Taiki).

The fieldwork was conducted in 7 municipalities in the Tokachi District, located in the eastern part of Hokkaido in northern Japan. We collected questing ticks throughout the district: two northern sites (Shikaoi (43°11′N, 142°59′E) and Ashoro (43°20′N, 143°36′E)), three central sites (Memuro (42°49′N, 142°58′E), Urahoro (42°46′N, 143°44′E) and Shimizu (42°59′N, 142°50′E)) and two southern sites (Hiroo (42°17′N, 143°16′E) and Taiki (42°31′N, 143°11′E)) (Fig. 1). Detailed geographical and climate features of the Tokachi District are described by Yazaki et al. (2013) and Fukushima et al. (2019). Between May and November 2017, field expeditions were performed once per month to collect questing ticks by the flagging method (Dantas-Torres et al., 2013). Microscopic observation was performed to determine the species and developmental stages (larva, nymph and adult) in accordance with the morphology of ticks. Ticks were identified using recognized morphological keys (Kitaoka, 1985; Yamaguti et al., 1971). Identified ticks were stored in 70% ethanol prior to DNA extraction.

### 2.2 DNA extraction

To avoid contamination, all steps were carried out on a clean bench. Throughout all manipulations, sterile filter tips were used. DNA extraction was processed individually or in pools for larvae, whereas nymph and adult ticks were processed individually. Identified larvae were pooled according to collection month, collection site, and species (2–10 individuals per pool). For *Ixodes ovatus*, one larva was tested. The ethanol-preserved ticks were rinsed twice with 70% ethanol and then immersed in distilled water (Yu et al., 2016). Ticks were homogenized in 75 μl of PBS using a PowerMasher II homogenizer (Nippi, Tokyo, Japan) and sterilized homogenization tube BioMasher II (Nippi). DNA was extracted using NucleoSpin Tissue (Macherey–Nagel, Düren, Germany) according to the manufacturerʼs instructions. DNA was eluted from columns with 50 μl of elution buffer. To confirm the purity of the eluates, the concentration of each DNA extract was assessed by Nanodrop spectrophotometers (Thermo Fisher Scientific, MA, USA). The extracted samples were stored at −30 °C until use.

### 2.3 PCR detection of *B. burgdorferi* (*s.l*.)

A nested PCR reaction was run on all DNA samples to detect *B. burgdorferi* (*s.l.*). Specific primers for *B. burgdorferi* (*s.l.*) (Table 1) were used to amplify the *flaB* gene encoding the flagellin protein (Yu et al., 2016). A 0.75 μl of DNA sample was added to 9.25 μl of reaction mixture that contained 1 μl of 10× PCR buffer, 1 μl of dNTPs, 0.2 μl of each primer (primers *flaB* outer primer F and *flaB* outer primer R, Table 1), 0.1 μl of Taq DNA polymerase (Blend Taq-Plus-; Toyobo, Osaka, Japan) and 6.75 μl of sterile Milli-Q water. The reaction conditions for the first PCR involved 4 min of pre-denaturation at 94 °C followed by 35 cycles of 30 s of denaturation at 94 °C, 30 s of annealing at 55 °C and extension at 72 °C for 1 min, and a final extension step at 72 °C for 10 min. The second PCR was performed in the same buffer using 0.5 μl of the first PCR products as a template and primers *flaB* nested primer F and *flaB* nested primer R (Table 1). The protocol for the second PCR was initial denaturation (94 °C, 4 min); followed by 35 cycles at 94 °C for 30 s, 58 °C for 30 s and 72 °C for 45 s, with the final extension step at 72 °C for 10 min. Amplicons of the second PCR were electrophoresed and stained with ethidium bromide as described previously (Yu et al., 2016). Detection of a band close to 411 bp under UV was considered positive.

**Table 1:** Oligonucleotide primers used in this study

Organism	Target gene	Oligonucleotide primer	Primer sequence (5′-3′)	Amplicon size (bp)	Reference
*B. burgdorferi* (*s.l.*)	*flaB*	Outer primer F	CTGCTGGCATGGGAGTTTCT	725	Yu et al. (2016)
		Outer primer R	TCAATTGCATACTCAGTACT		
		Nested primer F	GCAGTTCAATCAGGTAACGGC	411	Yu et al. (2016)
		Nested primer R	AGAAGGTGCTGTAGCAGGTG		
	*rrf* (5s)*-rrl* (23s)	23SN1	ACCATAGACTCTTATTACTTTGAC	373	Choi et al. (2007)
		23SC1	TAAGCTGACTAATACTAATTACCC		
		23SN2	ACCATAGACTCTTATTACTTTGACCA	227	Choi et al. (2007)
		5SCB	GAGAGTAGGTTATTGCCAGGG		
*Rickettsia* spp.	*gltA*	CS-78	GCAAGTATCGGTGAGGATGTAAT	401	Labruna et al. (2004)
		CS-323	GCTTCCTTAAAATTCAATAAATCAGGAT		
	16S rRNA	Rick_16S_F3	ATCAGTACGGAATAACTTTTA	1328	Anstead & Chilton (2013)
		Rick_16S_F4	TGCCTCTTGCGTTAGCTCAC		
		rrs2_seq_1	AGGCCTTCATCACTCACTCG^a^		Thu et al. (2019)
		rrs2_seq_2	CTACACGCGTGCTACAATGG^a^		
		R16S_Fw1	AGAAAAAGCCCCGGCTAACTC^a^		This study
		R16S_Rv1	CCATGCAACACCTGTGTGTG^a^		
	*ompB*	120_2788	AAACAATAATCAAGGTACTGT	816	Roux & Raoult (2000)
		120_3599	TACTTCCGGTTACAGCAAAGT		
	*sca4*	D1f	ATGAGTAAAGACGGTAACCT	928	Sekeyova et al. (2001)
		D928r	AAGCTATTGCGTCATCTCCG		

a Primers that were used only for the sequencing of amplicons.

The species of the *B. burgdorferi* (*s.l.*) complex detected in the samples were later identified through sequencing of the amplicons of the second PCR. When the *flaB* sequence did not allow a precise identification, the *rrf* (5S)-*rrl* (23S) ribosomal RNA (*5S–23S*) gene was amplified and sequenced for confirmation of the *B. burgdorferi* (*s.l.*) species. The primer sets used are shown in Table 1. PCR conditions were the same as previously reported (Choi et al., 2007).

### 2.4 PCR detection of *Rickettsia* spp.

PCR preparation was carried out in sterile conditions as described above. All of the tick DNA samples were subjected to PCR targeting *gltA* as a screening test for *Rickettsia* spp. DNA. Primers and PCR conditions have been described previously (de Sousa et al., 2018, Table 1). One microliter of DNA sample was added to a 9-μl reaction mixture that contained 1 μl of 10× PCR buffer, 1 μl of dNTPs, 0.2 μl of each primer set (primers CS-78 and CS-323, Table 1), 0.1 μl of Taq DNA polymerase (Blend Taq-Plus-; Toyobo) and 6.5 μl of sterile Milli-Q water. Detection of a band around 401 bp under UV was considered positive. Additional PCR assays were performed based on three genes: 16S ribosomal RNA gene (16S rRNA), outer membrane protein B gene (*ompB*), and surface cell antigen-4 (*sca4*) gene. The primer sets used for each reaction are shown in Table 1. PCR conditions were the same as previously reported by Thu et al. (2019) except for the annealing temperatures for *ompB* and *sca4* PCR (54 °C for *ompB* PCR and 56 °C for *sca4* PCR).

The *Rickettsia* species detected in the samples were identified in two steps. First, *gltA* genotyping was performed through sequencing of the amplicons of *gltA* PCR. Then, selected-sample representatives of the *gltA* genotype were submitted to further characterization of *Rickettsia* spp. through sequencing of 16S rRNA, *ompB* and *sca4* genes.

### 2.5 Sequencing

The amplified PCR products were purified using a NucleoSpin Gel and PCR Clean-up kit (Macherey-Nagel). Each PCR product was subjected to DNA sequencing (Fasmac, Kanagawa, Japan), and ClustalW was used to align the sequences. The newly generated DNA sequences were submitted to the DNA Data Bank of Japan (DDBJ) (http://www.ddbj.nig.ac.jp) under the accession numbers: LC496815-LC496832 (*Borrelia flaB*); LC496811-LC496814 (*Rickettsia gltA*); MT378425-MT378437 (*Rickettsia* 16S rRNA); LC544128-LC544135 (*Rickettsia ompB*); and LC544136-LC544138 (*Rickettsia sca4*). Inter-species comparison of sequences based on BLASTn was performed using NCBI software Megablast (https://blast.ncbi.nlm.nih.gov/Blast.cgi). The nucleotide collection (nr/nt) database, which links GenBank, EMBL, DDBJ, PDB and RefSeq sequences was used.

### 2.6 Phylogenetic analysis

DNA sequences obtained were aligned with sequences of representative *Borrelia* spp. or *Rickettsia* spp. using ClustalW 1.6 as implemented in MEGA 7 (Kumar et al., 2016). After manual confirmation, phylogenetic trees were constructed using the maximum likelihood method according to the Tamura 3-parameter (Tamura, 1992) model with bootstrap tests of 1000 replicates using MEGA 7.

### 2.7 Statistical tests

Microorganism prevalence in the individual tick DNA samples and their 95% confidence intervals (95% CI) were calculated using the GraphPad Prism software (GraphPad Software, San Diego, CA, USA). Microorganism prevalence in larvae was estimated by taking into account the number of pools and the number of larvae per pool. We assumed a maximum positive rate if all larvae of a positive pool were infected and a minimum positive rate if only one larva in a positive pool was infected.

## 3 Results

### 3.1 Tick collection

In total, 1563 ticks (599 adults, 191 nymphs and 773 larvae) were collected between May and November 2017 from 7 sites in Tokachi District. The number of ticks, tick species, and developmental stages collected varied based on sampling month and site. Four species of ticks were identified based on morphological criteria: *I. ovatus* (*n* = 364), *Ixodes persulcatus* (*n* = 296), *Haemaphysalis japonica* (*n* = 143) and *Haemaphysalis megaspinosa* (*n* = 760). *Ixodes persulcatus* was found in all sites examined across Tokachi District. The detailed features of the tick samples (sampling month, sampling sites, species and developmental stage) are presented in Supplementary Table S1. The 1563 live ticks that were collected by flagging, was divided into two sets. One set was allocated to laboratory rearing in other experiments (Umemiya-Shirafuji et al., unpublished data) and the remaining samples were submitted to DNA extraction: a total of 1155 ticks were used for DNA extraction and microorganism detection in the present study. Overall, 594 DNA samples including 527 individual tick DNA samples and 67 pooled larval tick DNA samples were prepared. The individual tick DNA samples were prepared from 295 *I*. *ovatus* (123 males; 171 females; and 1 larva), 169 *I*. *persulcatus* (72 males; 77 females; and 20 nymphs), 18 *H*. *japonica* (6 males; 1 female; 10 nymphs; and 1 larva), and 45 *H*. *megaspinosa* (26 males; 8 females; and 11 nymphs). The pooled tick DNA samples were prepared from larval stages of *I*. *persulcatus* (61; 9 pools) and *H*. *megaspinosa* (567; 58 pools) (Supplementary Table S1).

### 3.2 Detection of *B. burgdorferi* (*s.l*.) in ticks

*Borrelia burgdorferi* (*s.l.*)-positive ticks were detected in 6 (Memuro, Shikaoi, Urahoro, Shimizu, Taiki and Ashoro) of the 7 sites surveyed (Supplementary Table S1). DNA fragments of *Borrelia* spp. were detected in *I*. *ovatus* and *I*. *persulcatus* in the present study. The overall prevalence of *B. burgdorferi* (*s.l.*) was 21.8% (64/294; 95% CI: 17.2–26.9%) in *I*. *ovatus* (8/123 males; 56/171 females). One larva of *I*. *ovatus* tested was negative. The prevalence in *I*. *persulcatus* was 23.7% (40/169; 95% CI: 17.5–30.8%) and positive ticks included adults (12/72 males; 26/77 females,) and nymphs (2/20). All larval pools were negative (0/9 pools).

### 3.3 *flaB* genotyping and species classification of *B. burgdorferi* (*s.l*.)

**Fig. 2:**
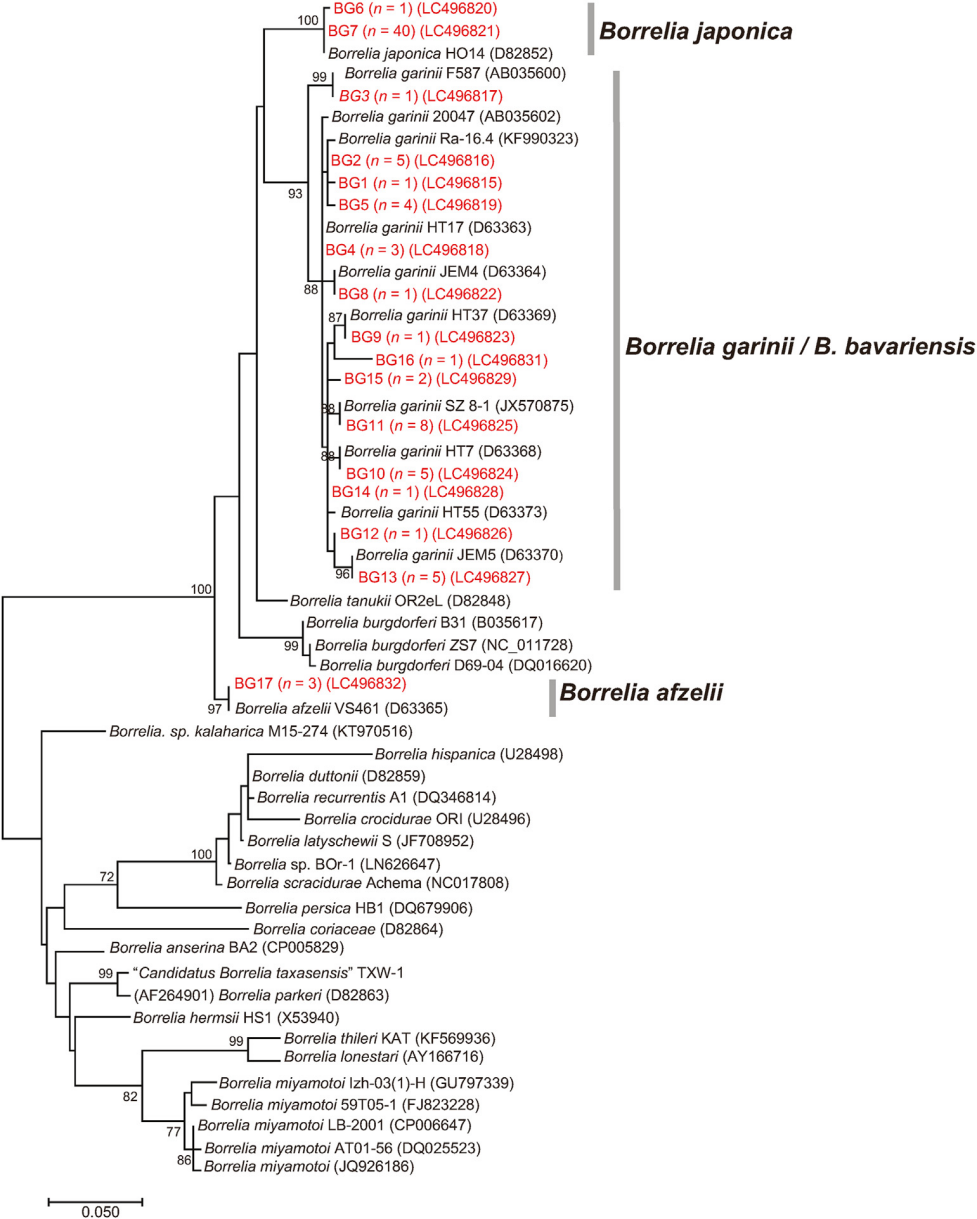
Phylogenetic tree based on the sequences of the *flaB* gene (321 bp) for *Borrelia* spp. Seventeen different *flaB* genotypes (BG1 to BG17) were identified in the present study (red-colored letters). Numbers in parentheses represent GenBank accession numbers. The unit of branch length is nucleotide substitutions per site.

Of the 104 samples that tested positive for *B. burgdorferi* (*s.l.*), 83 *flaB* PCR amplicons were successfully sequenced. The species identity of the remaining 21 samples was confirmed by sequencing the *5S–23S* gene. From analyzing the 83 *flaB* sequences, 17 different *flaB* genotypes (referred to as BG1 to BG17) were identified. Fifteen genotypes were detected in *I*. *persulcatus* (BG1, BG2, BG3, BG4, BG5, BG8, BG9, BG10, BG11, BG12, BG13 BG14, BG15, BG16 and BG17) whereas the remaining two (BG6 and BG7) were only found in *I*. *ovatus*. The *flaB* sequences obtained in this study shared 99 or 100% identity with the closest *Borrelia* species sequences available in the GenBank database (Table 2). In the phylogenetic tree inferred from *flaB* analysis, 14 genotypes (BG1, BG2, BG3, BG4, BG5, BG8, BG9, BG10, BG11, BG12, BG13 BG14, BG15 and BG16) were found in the same clade with *B. garinii* and the closely related *Borrelia bavariensis*. The BLASTn analysis of the *5S–23S* gene sequences obtained from the corresponding samples (data not shown) identified some of these genotypes as *B. garinii* and others as *B. bavariensis*. The 14 genotypes were therefore classified as *B. garinii/B. bavariensis* (Fig. 2). BG6 and BG7 formed a distinct cluster with *Borrelia japonica* and were identified as *B. japonica*. The *5S–23S* gene sequences (data not shown) also confirmed the presence of *B. japonica* in the samples from which BG6 and BG7 were amplified. BG17 clustered with *B. afzelii* (Fig. 2).

**Table 2:** Percentage of identity of *flaB* sequences of this study with sequences of the closest *Borrelia* species by BLAST analysis

BG No.	Tick ID	Tick species	Developmental stage	% Identity of *flaB* with the closest *Borrelia* spp. (GenBank ID)
BG1 (*n* = 1)	Ipers20170036	*I. persulcatus*	Female	99% with *B. garinii* (MK604262)
BG2 (*n* = 5)	Iper20170048	*I. persulcatus*	Female	99% with *B. garinii* (MN193533)
BG3 (*n* = 1)	Iper20170143	*I. persulcatus*	Nymph	100% with *B. garinii* (AB035600)
BG4 (*n* = 3)	Iper20170022	*I. persulcatus*	Female	100% with *B. garinii* (D63363)
BG5 (*n* = 4)	Iper20170037	*I. persulcatus*	Female	99% with *B. garinii* (MK604263)
BG6 (*n* = 1)	Iovat20170110	*I. ovatus*	Female	99% with *B. japonica* (D82852)
BG7 (*n* = 40)	Iovat20170021	*I. ovatus*	Female	100% with *B. japonica* (D82852)
BG8 (*n* = 1)	Iper20170112	*I. persulcatus*	Male	100% with *B. garinii* (D63364)
BG9 (*n* = 1)	Iper20170102	*I. persulcatus*	Male	100% with *B. garinii* (D63369)
BG10 (*n* = 5)	Iper20170054	*I. persulcatus*	Female	100% with *B. garinii* (D63368)
BG11 (*n* = 8)	Iper20170057	*I. persulcatus*	Female	100% with *B. garinii* (JX570875^a^)
BG12 (*n* = 1)	Iper20170040	*I. persulcatus*	Female	100% with *B. garinii* (KU672560)
BG13 (*n* = 5)	Iper20170071	*I. persulcatus*	Female	100% with *B. garinii* (D63370)
BG14 (*n* = 1)	Iper20170073	*I. persulcatus*	Female	100% with *B. garinii* (KU672560)
BG15 (*n* = 2)	Iper20170072	*I. persulcatus*	Female	100% with *B. garinii* (AB001714)
BG16 (*n* = 1)	Iper20170101	*I. persulcatus*	Male	99% with *B. garinii* (KT356614)
BG17 (*n* = 3)	Iper20170039	*I. persulcatus*	Female	100% with *B. afzelii* (MT007941)

*Abbreviation*: BG, *flaB* genotype of *Borrelia* spp.

a JX570875 is registered in the GenBank as *Borrelia garinii* strain SZ 8-1. However, recent findings (see http://borreliabase.org/) indicate that *Borrelia garinii* strain SZ is rather *B. bavariensis*, a species closely related to *Borrelia garinii*.

### 3.4 Detection of *Rickettsia* spp. in ticks

Ticks positive for *Rickettsia* spp. were detected in 6 (Memuro, Shikaoi, Urahoro, Hiroo, Taiki and Ashoro) out of the 7 provinces (Supplementary Table S1). Tick samples that showed positivity for *Rickettsia* spp. *gltA* included adults, nymphs and larvae of *I*. *persulcatus* and *H*. *megaspinosa*. All of the *I*. *ovatus* and *H*. *japonica* samples were negative. The overall prevalence of *Rickettsia* spp. was 41.4% (70/169; 95% CI: 34.5–49.8%) in *I*. *persulcatus* adults and nymphs (21/72 males; 44/77 females; 5/20 nymphs). Most of the *I*. *persulcatus* larval pools were positive (8/9), and the maximum and minimum positive rates among the larvae were estimated at 90.2% (55/61) and 13% (8/61), respectively. Among *H. megaspinosa* ticks the prevalence was 11.1% (5/45; 95% CI: 3.7–24.1%) for adults and nymphs (3/26 males; 2/8 females; 0/11 nymphs). Three out of the 58 larval pools were positive, suggesting 5.29% (30/567) and .53% (3/567) for the maximum and minimum positive rates among the *H. megaspinosa* larvae.

### 3.5 *gltA* genotyping and species classification of *Rickettsia* spp.

PCR amplicons of 86 samples found positive for *Rickettsia* spp. were sequenced and differentiated into 4 *gltA* genotypes (RG1, RG2, RG3 and RG4). RG1 was obtained from *I*. *persulcatus* (adult, nymph and larva) and a larval pool of *H*. *megaspinosa*. RG2 and RG4 were recovered from *I*. *persulcatus* (adult, nymph and larva) whereas RG3 was detected in *H*. *megaspinosa* (adult and larva). In the multiple gene sequencing of samples representing the *gltA* genotypes, the 16S rRNA gene was successfully amplified for all genotypes. The *ompB* gene was amplified in all samples of 3 genotypes (RG1, RG3 and RG4) except for *H. megaspinosa* (Hmega2017 Hiroo L007; RG1). The *sca4* gene, however, was successfully amplified only for RG3. The *gltA* sequences obtained in this study shared 98–100% identity with the closest published *Rickettsia* species sequence (Table 3).

**Table 3:** Results of the BLAST analysis of the *Rickettsia* spp. *gltA*, 16S rRNA, *ompB*, and *sca4* sequences obtained in this study

RG No.	Tick ID	Tick species and developmental stage	% Identity with the closest *Rickettsia* species (GenBank ID)			
			*gltA*	16S rRNA	*ompB*	*sca4*
RG1 (*n* = 50)	Ipers20170019	*I. persulcatus* (male)	100% with *R. helvetica* (KU310588)	100% with *R. helvetica* (MH618376)	99% with *R. helvetica* (MF163037)	na
	Ipers20170021	*I. persulcatus* (female)	100% with *R. helvetica* (KU310588)	100% with *R. helvetica* (MH618376)	99% with *R. helvetica* (MF163037)	na
	Ipers20170135	*I. persulcatus* (nymph)	100% with *R. helvetica* (KU310588)	100% with *R. helvetica* (MH618376)	99% with *R. helvetica* (MF163037)	na
	Ipers2017 Shikaoi L001	*I. persulcatus* (larva)	100% with *R. helvetica* (KU310588)	100% with *R. helvetica* (MH618376)	99% with *R. helvetica* (MF163037)	na
	Hmega2017 Hiroo L007	*H. megaspinosa* (larva)	100% with *R. helvetica* (KU310588)	98% with *R. raoultii* (MK304546) 98% with *R. conorii* (NR_074480) 98% with *R. helvetica* (MH618376)	na	na
RG2 (*n* = 28)	Ipers20170103	*I. persulcatus* (male)	100% with “*Ca*. R. tarasevichiae” (MN450397)	100% with “*Ca*. R. tarasevichiae” (MN446745)	na	na
	Ipers20170025	*I. persulcatus* (female)	100% with “*Ca*. R. tarasevichiae” (MN450397)	100% with “*Ca*. R. tarasevichiae” (MN446745)	na	na
	Ipers20170184	*I. persulcatus* (nymph)	100% with “*Ca*. R. tarasevichiae” (MN450397)	100% with “*Ca*. R. tarasevichiae” (MN446745)	na	na
	Ipers2017 Taiki L001	*I. persulcatus* (larva)	100% with “*Ca*. R. tarasevichiae” (MN450397)	100% with “*Ca*. R. tarasevichiae” (MN446745)	na	na
RG3 (*n* = 7)	Hmega20170010^a^	*H. megaspinosa* (male)	100% with “*Ca*. R. principis” (AY578115)	99% with *R. raoultii* (MK304546) 99% *R. conorii* (NR_074480)	100% with “*Ca*. R. principis” (MG544987)^b^	99% with *R. heilongjiangensis* (AP019865)
	Hmega20170004^a^	*H. megaspinosa* (female)	100% with “*Ca*. R. principis” (AY578115)	99% with *R. raoultii* (MK304546) 99% *R. conorii* (NR_074480)	100% with “*Ca*. R. principis” (MG544987)^b^	98% with *R. heilongjiangensis* (AP019865)
	Hmega2017 Hiroo L009^a^	*H. megaspinosa* (larva)	100% with “*Ca*. R. principis*”* (AY578115)	99% with *R. raoultii* (MK304546) 99% with *R. conorii* (NR_074480)	100% with “*Ca*. R. principis” (MG544987)^b^	98% with *R. heilongjiangensis* (AP019865)
RG4 (*n* = 1)	Ipers20170166	*I. persulcatus* (male)	99% with “*Ca*. R. tarasevichiae” (MN450397) 89% with *R. helvetica* (KU310588)	100% with *R. helvetica* (MH618376)	99% with *R. helvetica* (MF163037)	na

*Abbreviations*: *Ca*., *Candidatus*; na, not amplified; RG, *gltA* genotype of *Rickettsia* spp.

a Identities with “*Ca*. R. principis” 16S rRNA and *sca4* are not shown due to absence of reference sequences in the database.

b Query coverage was 95%.

**Fig. 3:**
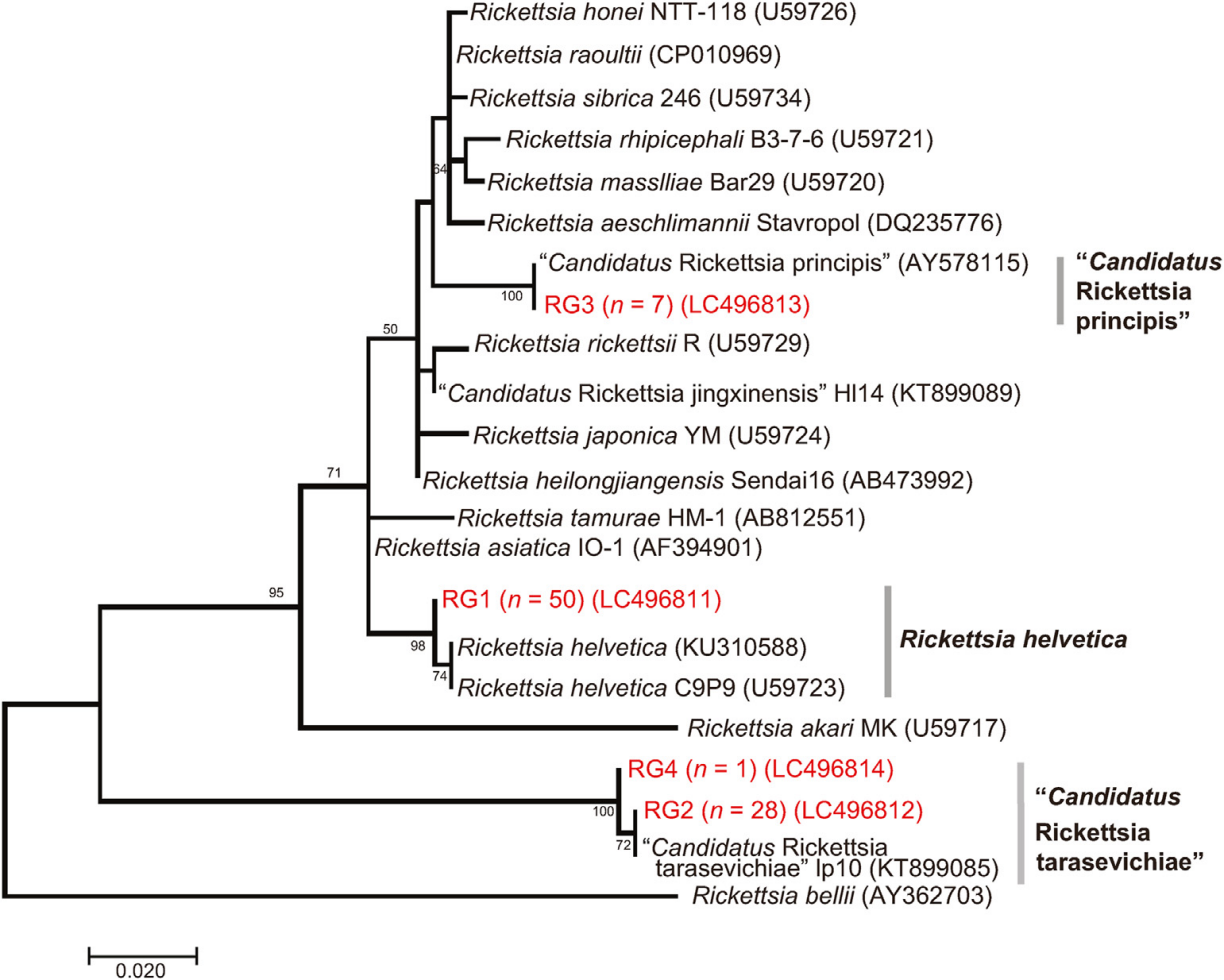
Phylogenetic tree based on the sequences of the *gltA* gene (332 bp) for *Rickettsia* spp. Amplicons by PCR for *Rickettsia gltA* were sequenced and classified into 4 different *gltA* genotypes (RG1 to RG4). DNA sequences obtained in the present study are indicated in red. Numbers in parentheses represent GenBank accession numbers. The unit of branch length is nucleotide substitutions per site.

**Fig. 4:**
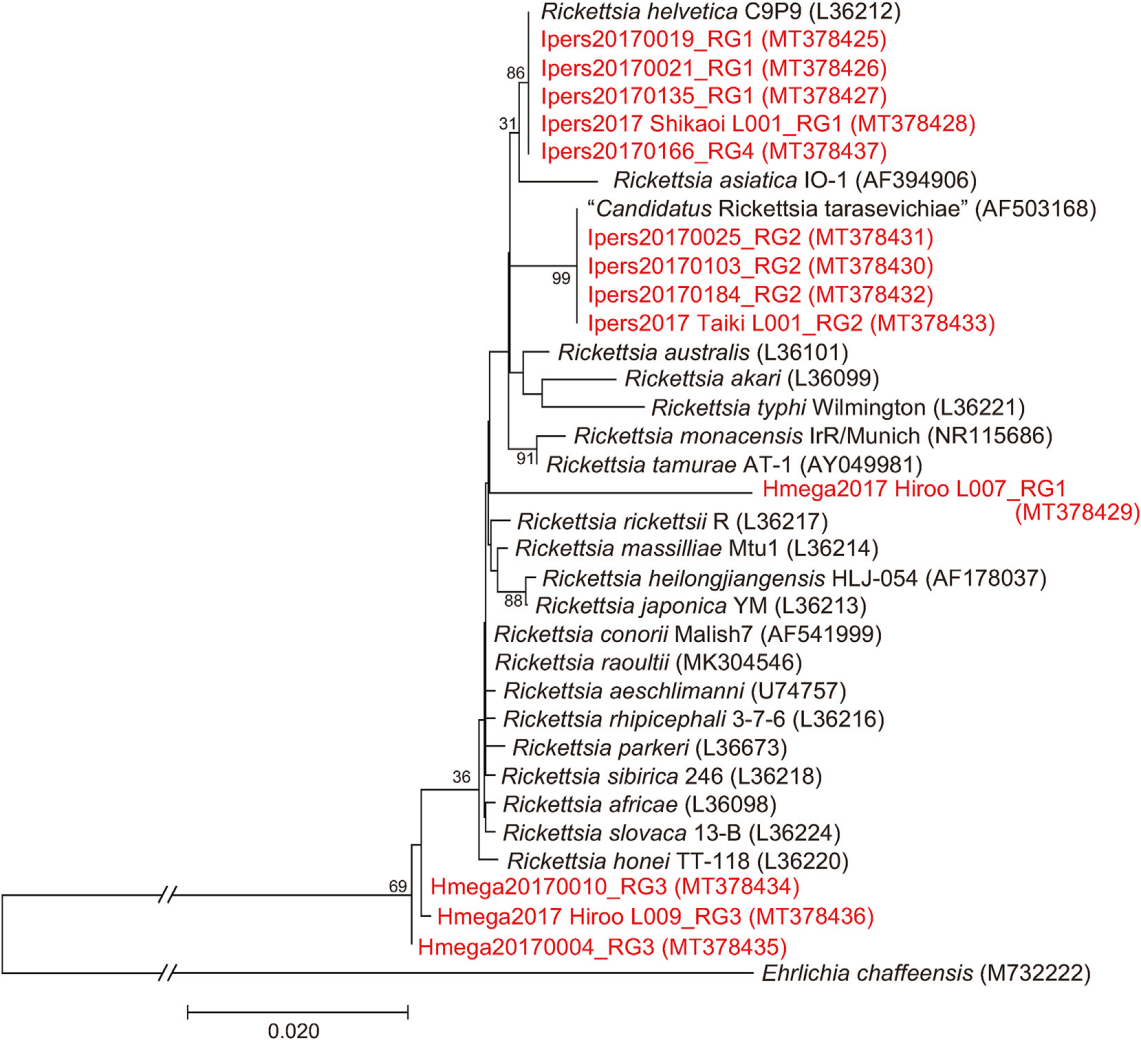
Phylogenetic tree based on the *Rickettsia* 16S rRNA gene (1179 bp). The tree was constructed using the maximum likelihood method with the Kimura 2-parameter model. The analysis was performed with bootstrap tests of 1000 replicates. DNA sequences obtained in the present study are indicated in red-highlighted-tick ID. Numbers in parentheses are GenBank accession numbers. The units of branch length are nucleotide substitutions per site. The long branches are shortened and presented as interrupted branches (see full-size image in Supplementary Fig. S1).

**Fig. 5:**
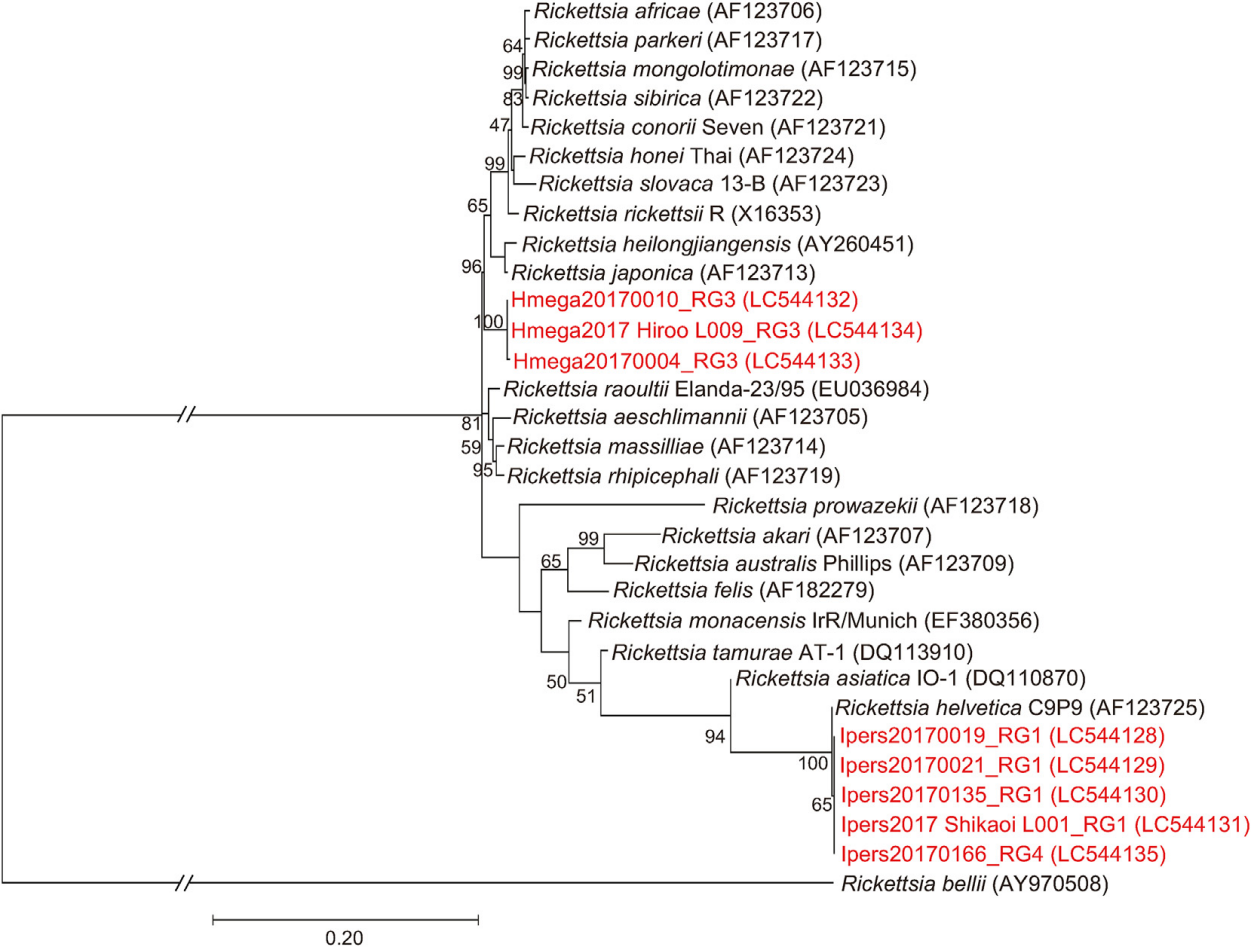
Phylogenetic tree based on the *Rickettsia ompB* gene (815 bp). The tree was constructed using the maximum likelihood method with the Tamura 3-parameter model. The analysis was performed with bootstrap tests of 1000 replicates. DNA sequences obtained in the present study are indicated in red-highlighted-tick ID. Numbers in parentheses represent *GenBank accession numbers*. The units of branch length are nucleotide substitutions per site. The long branches are shortened and presented as interrupted branches (see full-size image in Supplementary Fig. S2).

**Fig. 6:**
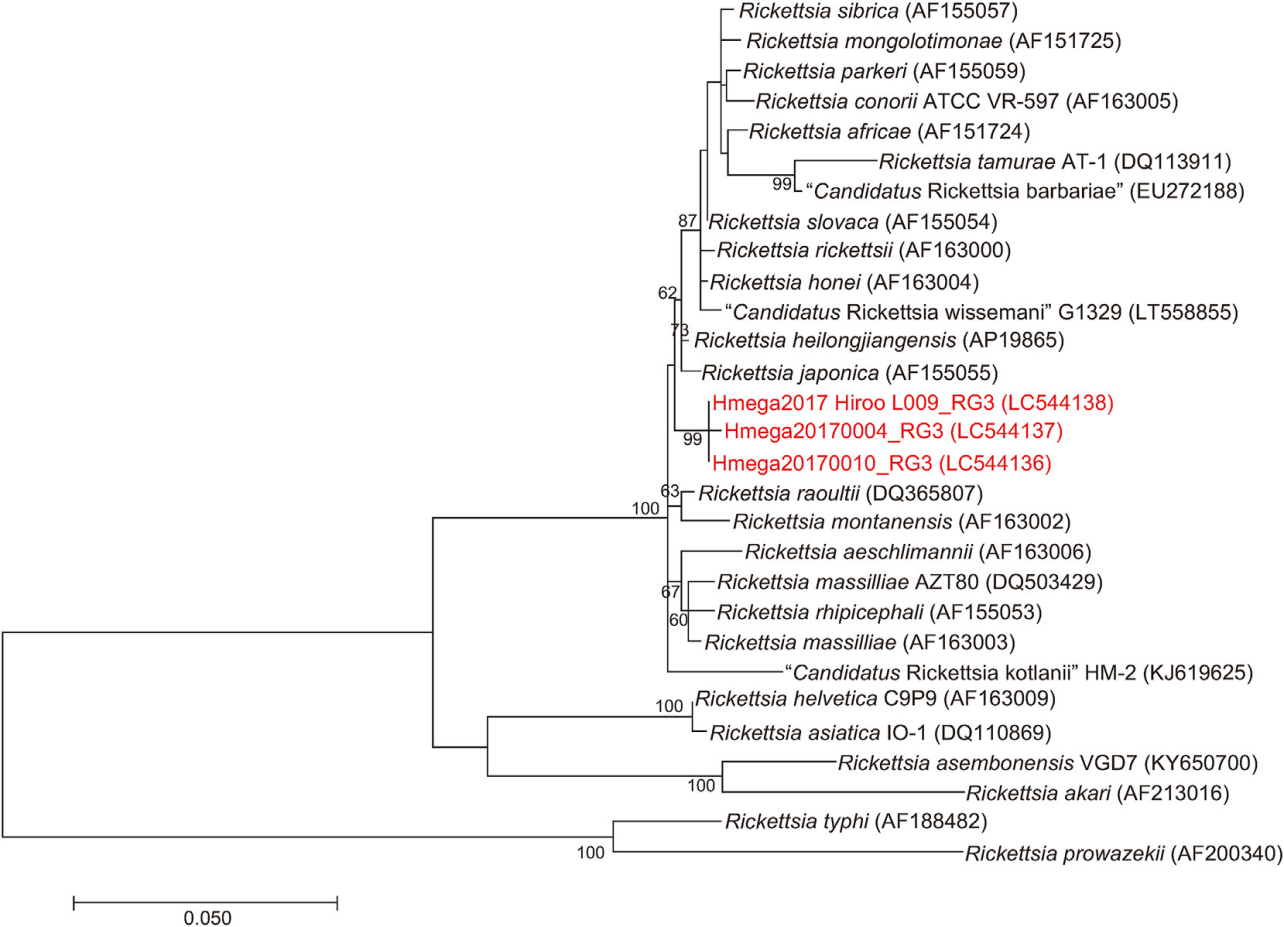
Phylogenetic tree based on the *Rickettsia sca4* (847 bp) gene. The tree was constructed using the maximum likelihood method with the Time reversible model. The analysis was performed with bootstrap tests of 1000 replicates. DNA sequences obtained in the present study are indicated in red-highlighted-tick ID. Numbers in parentheses are GenBank accession numbers. The units of branch length are nucleotide substitutions per site.

Phylogenetic trees inferred from *gltA* (Fig. 3), 16S rRNA (Fig. 4), *ompB* (Fig. 5) and *sca4* (Fig. 6) sequences were constructed using the data from our study and public sequences of validated *Rickettsia* species. Four out of the five representatives of the RG1 genotype are close to *R*. *helvetica* and formed a distinct cluster with *R*. *helvetica* in the *gltA* and *ompB* phylogenetic trees. One RG1 obtained from a larval pool of *H*. *megaspinosa* was located in the *R*. *helvetica gltA* cluster but formed a divergent branch in the 16S rRNA phylogenetic tree (*ompB* and *sca4* could not be amplified for that sample; Table 3). RG2 species identity was assessed using *gltA* and 16S rRNA phylogenetic trees, both of which located the genotype in a cluster with “*Ca*. R. tarasevichiae”. The RG3 genotype was classified as “*Ca*. R. principis” in the *gltA* phylogenetic tree. However, in the 16S rRNA, *ompB* and *sca4* phylogenetic trees, RG3 sequences formed a cluster distinct from other validated *Rickettsia* species. RG4 was identified in the “*Ca*. R. tarasevichiae” cluster of the *gltA* phylogenetic tree, whereas in the 16S rRNA and *ompB* trees, it was located within the cluster of *R*. *helvetica*.

Altogether, the BLAST and phylogenetic analyses based on the *gltA,* 16S rRNA, *ompB* and *sca4* sequences, showed that the *Rickettsia* spp. detected in the samples include species related to *R. helvetica*, “*Ca.* R. principis*”*, and “*Ca*. R. tarasevichiae”.

### 3.6 Distribution of *B. burgdorferi* (*s.l.*) and *Rickettsia* species

The presence of *Borrelia* spp. and *Rickettsia* spp. in tick samples collected from Tokachi District in 2017 is summarized in Table 4. *Borrelia japonica* was only detected in adult *I*. *ovatus* samples with a prevalence of 21.8% (64/294; 95% CI: 17.2–26.9%). *Borrelia garinii/B. bavariensis* was detected in both adult and nymphal *I*. *persulcatus* at a prevalence of 21.9% (37/169; 95% CI: 15.9–28.9%). *Borrelia afzelii* was found only in adult *I*. *persulcatus* at a prevalence of 1.8% (3/169; 95% CI: 0.4–5.1%). Concerning *Rickettsia* species, *R*. *helvetica* was detected at a prevalence of 26.0% (44/169; 95% CI: 19.6–33.3%) in adult and nymphal *I*. *persulcatus* and in 55.6% (5/9) of the larval pools. Among the *H. megaspinosa* larval pools, one (1.7%; 1/58) was positive for *R*. *helvetica*. The prevalence of “*Ca.* R. tarasevichiae” was 15.4% (26/169; 95% CI: 10.3–21.7%) in *I. persulcatus* adults and nymphs and 33.3% (3/9) in the larval pools. “*Candidatus* R. principis” was related to *H*. *megaspinosa* with a prevalence of 11.1% (5/45; 95% CI: 3.7–24.1%) and 3.4% (2/58) for adults and nymphs, and larval pools, respectively. The prevalence of these microorganisms in female ticks appeared to be higher than the values recorded in male ticks (Table 4). However, larger sample sizes will be needed to compare statistically the prevalence of the microorganisms between female and male ticks. The distribution of *Borrelia* and *Rickettsia* species varied across the sampling sites (Supplementary Table S1). *Rickettsia helvetica* and “*Ca*. R. tarasevichiae” found in 6 sites (Memuro, Shikaoi, Urahoro, Hiroo, Taiki and Ashoro) were the most widely distributed, followed by *B. japonica* (Memuro, Urahoro, Shimizu, Taiki and Ashoro) and *B. garinii/B. bavariensis* (Memuro, Shikaoi and Urahoro). “*Candidatus* R. principis” and *B. afzelii* were found in 2 (Hiroo, Taiki) and 1 (Memuro) site, respectively.

**Table 4:** Molecular detection of *Borrelia burgdorferi* (*s.l*.) and *Rickettsia* spp. among tick species collected in Tokachi district, Japan

Tick species	Stage^a^	*Borrelia burgdorferi* (*s.l*.) (%)			*Rickettsia* spp. (%)		
		*B. j.*	*B. g.*/*B. b.*	*B. a.*	*R. h.*	“*Ca.* R. t.”	“*Ca.* R. p.”
*I. ovatus*	Male	8/123 (6.5)	0/123 (0)	0/123 (0)	0/123 (0)	0/123 (0)	0/123 (0)
	Female	56/171 (32.7)	0/171 (0)	0/171 (0)	0/171 (0)	0/171 (0)	0/171 (0)
	Larva	0/1 (0)	0/1 (0)	0/1 (0)	0/1 (0)	0/1 (0)	0/1 (0)
*I. persulcatus*	Male	0/72 (0)	11/72 (15.3)	1/72 (1.4)	10/72 (13.9)	11/72 (15.3)	0/72 (0)
	Female	0/77 (0)	24/77 (31.2)	2/77 (2.6)	30/77 (39.0)	14/77 (18.2)	0/77 (0)
	Nymph	0/20 (0)	2/20 (10.0)	0/20 (0)	4/20 (20.0)	1/20 (5.0)	0/20 (0)
	Larva^b^	0/9 (0)	0/9 (0)	0/9 (0)	5/9 (56.0)	3/9 (33.0)	0/9 (0)
*H. megaspinosa*	Male	0/26 (0)	0/26 (0)	0/26 (0)	0/26 (0)	0/26 (0)	3/26 (12.0)
	Female	0/8 (0)	0/8 (0)	0/8 (0)	0/8 (0)	0/8 (0)	2/8 (25.0)
	Nymph	0/11 (0)	0/11 (0)	0/11 (0)	0/11 (0)	0/11 (0)	0/11 (0)
	Larva^b^	0/58 (0)	0/58 (0)	0/58 (0)	1/58 (1.7)	0/58 (0)	2/58 (3.4)
*H. japonica*	Male	0/6 (0)	0/6 (0)	0/6 (0)	0/6 (0)	0/6 (0)	0/6 (0)
	Female	0/1 (0)	0/1 (0)	0/1 (0)	0/1 (0)	0/1 (0)	0/1 (0)
	Nymph	0/10 (0)	0/10 (0)	0/10 (0)	0/10 (0)	0/10 (0)	0/10 (0)
	Larva	0/1 (0)	0/1 (0)	0/1 (0)	0/1 (0)	0/1 (0)	0/1 (0)

*Abbreviations*: *B*. *a*., *Borrelia afzelii*; *B. b.*, *B*. *bavariensis*; *B*. *g*., *Borrelia garinii*; *B*. *j*., *Borrelia japonica*; “*Ca*. R. p.”, “*Candidatus* Rickettsia principis”; “*Ca*. R. t. ”, “*Candidatus* Rickettsia tarasevichiae”; *R*. *h*., *Rickettsia helvetica*.

a Developmental stages of ticks collected.

b Larvae of *I*. *persulcatus* and *H*. *megaspinosa* were pooled according to collection month and collection site (2–10 individuals per pool).

## 4 Discussion

In this study, four hard tick species namely *I*. *ovatus*, *I*. *persulcatus*, *H*. *japonica* and *H*. *megaspinosa* were collected in 2017 from Tokachi District, Japan. The identified tick species are consistent with previous surveys on tick species in Tokachi (Inokuma et al., 2007), suggesting that these species are still dominant hard ticks in this area. *Ixodes ovatus* and *I*. *persulcatus* are the main causative species of human tick bites in Hokkaido to Honshu, northern to central Japan (Natsuaki, 2021). Some human cases with *H. japonica* bites were found in Hokkaido and Honshu (Yamauchi et al., 2010; Yamauchi & Nakatani, 2016; Sasaki et al., 2021). Although the primary hosts of *H. megaspinosa* are large mammals (e.g. deer, boar and Japanese serow) (Yamaguti et al., 1971), human cases of tick bites by *H. megaspinosa* were reported in Honshu (Seishima et al., 2000; Tsunoda, 2004; Hasegawa et al., 2016). The present PCR assays using DNA samples of questing ticks revealed that *I. persulcatus* carried *B. garinii/B. bavariensis*, *B. afzelii*, *R. helvetica* and “*Ca.* R. tarasevichiae”, and that *I. ovatus* was associated with *B. japonica*. *Haemaphysalis megaspinosa* carried *R. helvetica* and “*Ca.* R. principis”, while none of the *H. japonica* ticks collected in the present study was positive for the microorganisms that were targeted.

Currently, the *B. burgdorferi* (*s.l.*) species complex consists of more than 20 pathogenic, potentially pathogenic, non-pathogenic and unknown pathogenic bacteria that utilize *Ixodes* ticks as vectors (Stanek & Strle, 2018; Margos et al., 2019). *Borrelia garinii*, *B. bavariensis* and *B. afzelii* are human pathogens and cause Lyme borreliosis, whereas the pathogenicity of *B. japonica* is unknown. *Borrelia garinii* and *B. bavariensis*, previously referred to as *B. garinii* genospecies, are closely related species sharing the same geographical distribution and tick vectors (Margos et al., 2019). Discrimination of these *Borrelia* species requires a multilocus genotyping approach including eight genes. In our study, a two-gene approach confirming the occurrence of both species was selected and the samples of *B. burgdorferi* (*s.l.*) showing sequence identity with any of these two species were classified as *B. garinii/B. bavariensis. Ixodes persulcatus* is a suspected vector for *B. garinii*, *B. bavariensis* and *B. afzelii* (Masuzawa et al., 2005; Margos et al., 2019; Eisen, 2020). These pathogens have previously been reported in *I. persulcatus* collected from Japan (Fukunaga et al., 1996; Masuzawa, 2004; Murase et al., 2012). Furthermore, *I*. *persulcatus* was considered as a primary tick vector for Lyme borreliosis in Hokkaido (Fukunaga et al., 1993; Murase et al., 2012). The presence of *B. garinii/B. bavariensis* and *B. afzelii* in *I*. *persulcatus* from Tokachi area relates therefore to the vector role of this tick and its importance in Lyme borreliosis transmission.

Despite sharing the same geographical range and vector, *B. garinii*, *B. bavariensis* and *B. afzelii* seem to have different relative frequency. Fukunaga & Hamase (1995) indicated that *B. garinii* was predominant among isolates obtained from Lyme borreliosis patients and ticks in Japan. Meanwhile, Murase et al. (2012) showed that *B. garinii* infection rate was higher than that of *B. afzelii* in *I*. *persulcatus* from Hokkaido. In accordance, in our study, 92.5% (37/40) of the pathogens found in the *B. burgdorferi* (*s.l.*)-positive *I*. *persulcatus* samples were identified as *B. garinii/B. bavariensis*, and the remaining three positive samples were identical to *B. afzelii*. However, the infection rate (21.9%; 95% CI: 15.9–28.9%) of *B. garinii/B. bavariensis* in *I*. *persulcatus* from Tokachi area was lower than the *B. garinii* infection rate (33.8%) obtained by Murase et al. (2012) in the same tick species but in another site in Hokkaido Prefecture. The infection rate of *B. afzelii* (1.8%; 95% CI: 0.4–5.1%) showed in our study was similar to the value (4.9%) obtained by Murase et al. (2012). These authors investigated a ranching farm where a confirmed case of Lyme borreliosis has been reported, whereas we did not collect ticks in such farm in our study. It might suggest that the positive rates of pathogens vary depending on the characteristics of the study areas. While *B. garinii*, *B. bavariensis* and *B. afzelii* are found in Europe and Asia, *B. japonica* seems to be restricted to Japan (Kawabata et al., 1993; Li et al., 1998). Previous studies indicated that *I. ovatus* carries only *B. japonica* (Kawabata et al., 1993; Masuzawa, 2004; Murase et al., 2012) and Nakao et al. (1994) indicated that the tick and *Borrelia* species are not related to Lyme borreliosis. The presence of *B. japonica* and not the other *B. burgdorferi* (*s.l.*) genospecies in *I. ovatus* collected in this study, therefore relates to this tick species not being a vector of Lyme borreliosis. *Borrelia japonica* prevalence in *I. ovatus* (21.8%; 64/294, 95% CI: 17.2–26.9%) was also similar to the values (19.5%; 8/41) obtained by Murase et al. (2012).

We did not find *I. persulcatus* larvae carrying *B. garinii/B. bavariensis* or *B. afzelii*. In the absence of transovarial transmission of *B. burgdorferi* (*s.l.*) in *I. persulcatus* (Nefedova et al., 2004), acquisition of *B. garinii*, *B. bavariensis* and *B. afzelii* by its vector obviously results from feeding on the blood of host animals. The principal reservoir host for the species of *Borrelia* transmitted by *I. persulcatus* immatures in Hokkaido is the wood mouse, *Apodemus speciosus ainu* (Nakao & Miyamoto, 1993a). The *Borrelia* isolated from *A. speciosus ainu* was formerly identified as *B. garinii* (currently probably *B. bavariensis*) (Kawabata et al., 1993; Margos et al., 2019). In contrast, the long-tailed shrew *Sorex unguiculatus* is a reservoir for *B. japonica* and also a host animal for *I. ovatus* immatures in Hokkaido (Nakao & Miyamoto, 1993b). Tokachi area is a sympatric region for *I. ovatus* and *I. persulcatus*. Although the host ranges of *I. ovatus* and *I. persulcatus* partially overlap for their immature stages (Nakao & Miyamoto, 1993b), our results and previous studies suggest that *Borrelia* species detected from questing adults of both tick species are clearly distinguishable (Table 4). Further experimental investigations such as xenodiagnosis are needed to test the vector competence of the two tick species for different *Borrelia* species and observe the effect of these *Borrelia* infections on tick physiology.

In the present study, *Rickettsia* species were detected not only in adults and nymphs, but also in larvae. Of the three *Rickettsia* spp. detected, *R. helvetica* and “*Ca*. R. tarasevichiae” are pathogenic to humans, whereas “*Ca*. R. principis”, pathogenicity remains unknown. *R. helvetica* was first reported in Europe (Parola et al., 1998). Its prevalence in Europe has been evidenced in France (Fournier et al., 2000) and then in Japan (Fournier et al., 2002; Noji et al., 2005). It is known that vectors of *R. helvetica* in Japan are *I. persulcatus*, *I. ovatus* and *Ixodes monospinosus* (Fournier et al., 2002; Ishiguro et al., 2008). In the present study, we detected *R. helvetica gltA* genotype in *I. persulcatus*, corresponding to the finding of Inokuma et al. (2007) and suggesting that *I. persulcatus* could be a vector of *R. helvetica* in Tokachi District. Furthermore, the detection of *R. helvetica* in questing *I. persulcatus* larvae suggests transovarial transmission, although contaminations or partial feeding could not be excluded. *Rickettsia* *helvetica gltA* genotype (RG1) was also found in one pool of *H. megaspinosa* larvae (Hmega2017 Hiroo L007) that included 10 individual larvae; however, the sequence of the 16S rRNA gene from this pool formed a different clade with *R. helvetica* (Fig. 4; Hmega2017 Hiroo L007 RG1 (MT378429)). Although such finding indicates that *H. megaspinosa* might play a role as a vector for *R. helvetica*, further research is needed to fully characterize the *Rickettsia* species/strain carried by these larvae.

“*Candidatus* R. tarasevichiae” was regarded as non-pathogenic until 2012, when five patients with recent tick bites sought treatment in a hospital of northeastern China and were found to be infected with this pathogen (Jia et al., 2013). More recently, a retrospective investigation in eastern central China found that 56 patients who showed severe fever with thrombocytopenia syndrome-like illnesses were infected with “*Ca*. R. tarasevichiae” (Liu et al., 2016). The identification of “*Ca*. R. tarasevichiae” in *I. persulcatus* in our study is in agreement with a previous study (Inokuma et al., 2007) in the same district. “*Candidatus* R. tarasevichiae” was detected not only in adults and nymphs but also in larvae, suggesting that transovarial transmission occurred under natural conditions. Among the samples in which “*Ca*. R. tarasevichiae” *gltA* was identified, one (RG4 (*n* = 1); Ipers20170166) showed 16S rRNA and *ompB* sequences that clustered with *R. helvetica* sequences (Fig. 4, Fig. 5). This may be explained by the tick being infected with both “*Ca*. R. tarasevichiae” and *R. helvetica*. On the other hand, the life-cycle and pathogenicity for humans of “*Ca*. R. principis” remain unknown (Mediannikov et al., 2006). This rickettsial agent has previously been detected in the ticks *Haemaphysalis flava* and *H. japonica* in Japan (Gaowa et al., 2013) and in *Haemaphysalis danieli* in China (Chahan et al., 2007). A recent survey of SFG rickettsiae in questing ticks in Japan identified a *Rickettsia* genotype that was obtained from adult *H. megaspinosa* and clustered in the same clade with “*Ca*. R. principis” (Thu et al., 2019). In our study, *Rickettsia gltA* genotype RG3 was detected in *H. megaspinosa* adults and larvae and located in “*Ca*. R. principis” clade. In the BLASTn search, the *ompB* sequences of RG3 showed 100% identity with previously published “*Ca*. R. principis” sequences, although the query coverage was 95%. Because published “*Ca*. R. principis*”* sequences were shorter (*ompB*) or not available (16S rRNA and *sca4*), RG3 sequences formed a cluster distinct from other validated *Rickettsia* species in the *ompB*, 16S rRNA and *sca4* phylogenetic trees. Based on BLASTn search (*gltA* and *ompB*) and *gltA* phylogenetic tree, RG3 was identified as genetically related to “*Ca*. R. principis”. These results support previous reports and also provide, to our knowledge, the first evidence of the occurrence of “*Ca*. R. principis” in larvae of *H. megaspinosa*. The detection of “*Ca*. R. principis” in questing *H. megaspinosa* larvae indicates transovarial transmission of “*Ca*. R. principis” in the tick species.

## 5 Conclusions

Our findings suggest that major risks of Lyme borreliosis and SFG rickettsiosis agents in the Tokachi District are currently restricted to *I*. *persulcatus*. It would be informative for farmworkers, hunters, local residents as well as tourists to make aware of infection risks associated with tick bites. Furthermore, the sampling design (sampling season and frequency) as well as tick species life-cycle patterns might explain the differences in tick species and tick life stage between sampling sites. In the future, annual or yearly surveys of ticks and their carrying microorganisms will be needed to reveal their seasonal occurrence in the region to constantly avoid the risk for tick-borne diseases.

## Funding

This study was supported by the 10.13039/501100001700Ministry of Education, Culture, Sports, Science and Technology of Japan as a project for Joint Usage/Research Center.

## Ethical approval

Not applicable.

## CRediT author statement

Kiyoshi Okado: methodology, validation, formal analysis, investigation, writing - original draft, visualization. Paul Franck Adjou Moumouni: methodology, validation, formal analysis, investigation, resources, writing - review and editing. Seung-Hun Lee: methodology, validation, formal analysis, investigation, writing - review and editing. Thillaiampalam Sivakumar: formal analysis, writing - review and editing. Naoaki Yokoyama: conceptualization, investigation, writing - review and editing, project administration. Kozo Fujisaki: conceptualization, writing - review and editing. Hiroshi Suzuki: conceptualization, investigation, writing - review and editing, project administration. Xuenan Xuan: conceptualization, investigation, resources, project administration, funding acquisition. Rika Umemiya-Shirafuji: conceptualization, investigation, resources, writing - review and editing, visualization, supervision, funding acquisition. All authors read and approved the final manuscript.

## Data availability

The newly generated DNA sequences were submitted to the DNA Data Bank of Japan (DDBJ) (http://www.ddbj.nig.ac.jp) under the accession numbers: LC496815-LC496832 (*Borrelia flaB*); LC496811-LC496814 (*Rickettsia gltA*); MT378425-MT378437 (*Rickettsia* 16S rRNA); LC544128-LC544135 (*Rickettsia ompB*); and LC544136-LC544138 (*Rickettsia sca4*).

## Declaration of competing interests

The authors declare that they have no known competing financial interests or personal relationships that could have appeared to influence the work reported in this paper.

## Appendix A Supplementary data

Multimedia component 1 **Supplementary Figure S1.** Full size image of Fig. 4. Multimedia component 1

Multimedia component 2 **Supplementary Figure S2.** Full size image of Fig. 5. Multimedia component 2

Multimedia component 3 **Supplementary Table S1.** Tick species distribution and prevalence of *Borrelia burgdorferi (s.l.)* and *Rickettsia* spp. among questing ticks collected in Tokachi District, eastern Hokkaido, Japan. Multimedia component 3
