# Supplementary figures and images for: Molecular detection of Borrelia burgdorferi (sensu lato) and Rickettsia spp. in hard ticks distributed in Tokachi District, eastern Hokkaido, Japan
Source: Curr Res Parasitol Vector Borne Dis. 2021 Nov 11;1:100059. doi: 10.1016/j.crpvbd.2021.100059 (PMC8906132; doi:10.1016/j.crpvbd.2021.100059)

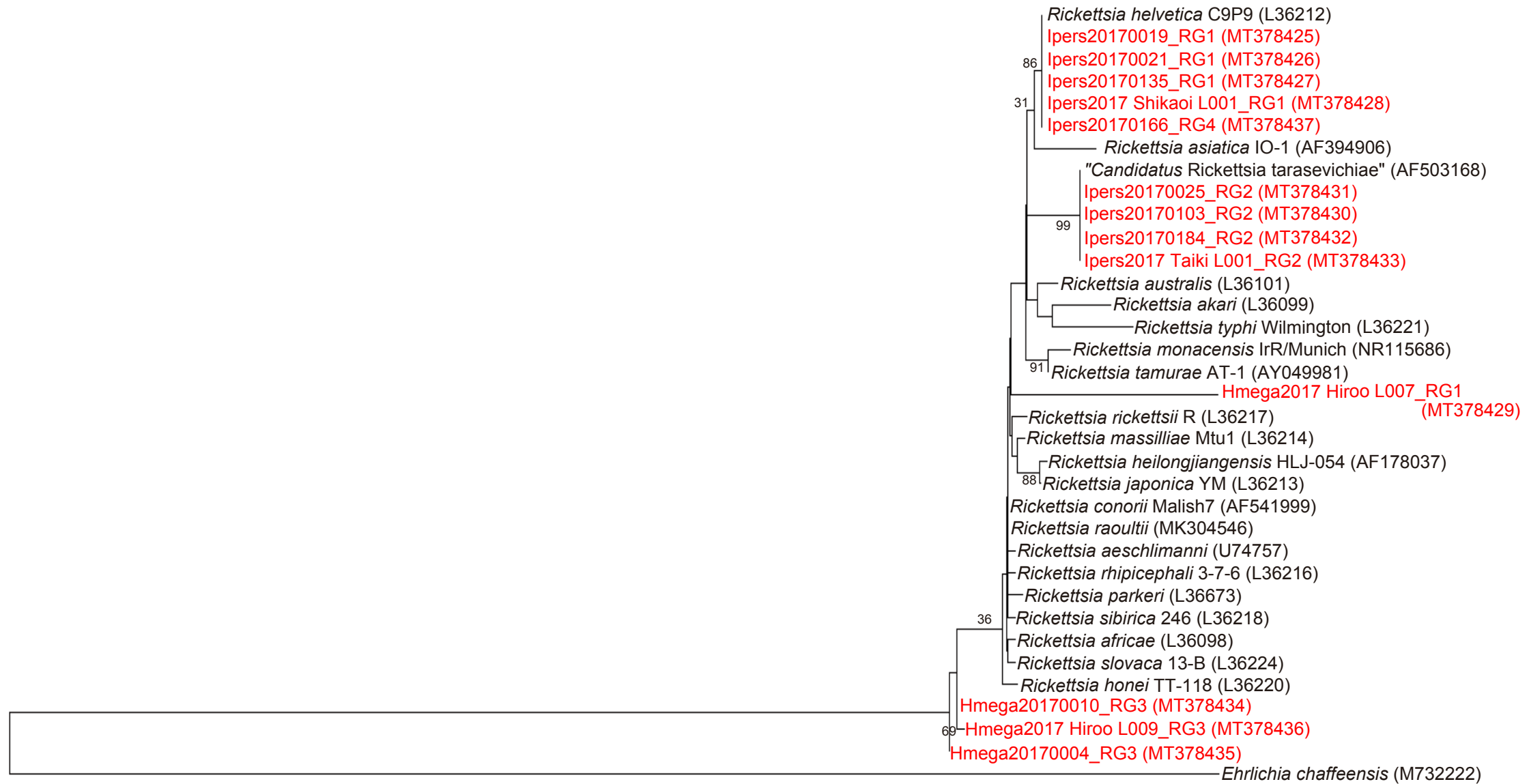

Supplement: Multimedia component 1 — Supplementary Figure S1. Full size image of Fig. 4. [file mmc1.pdf]

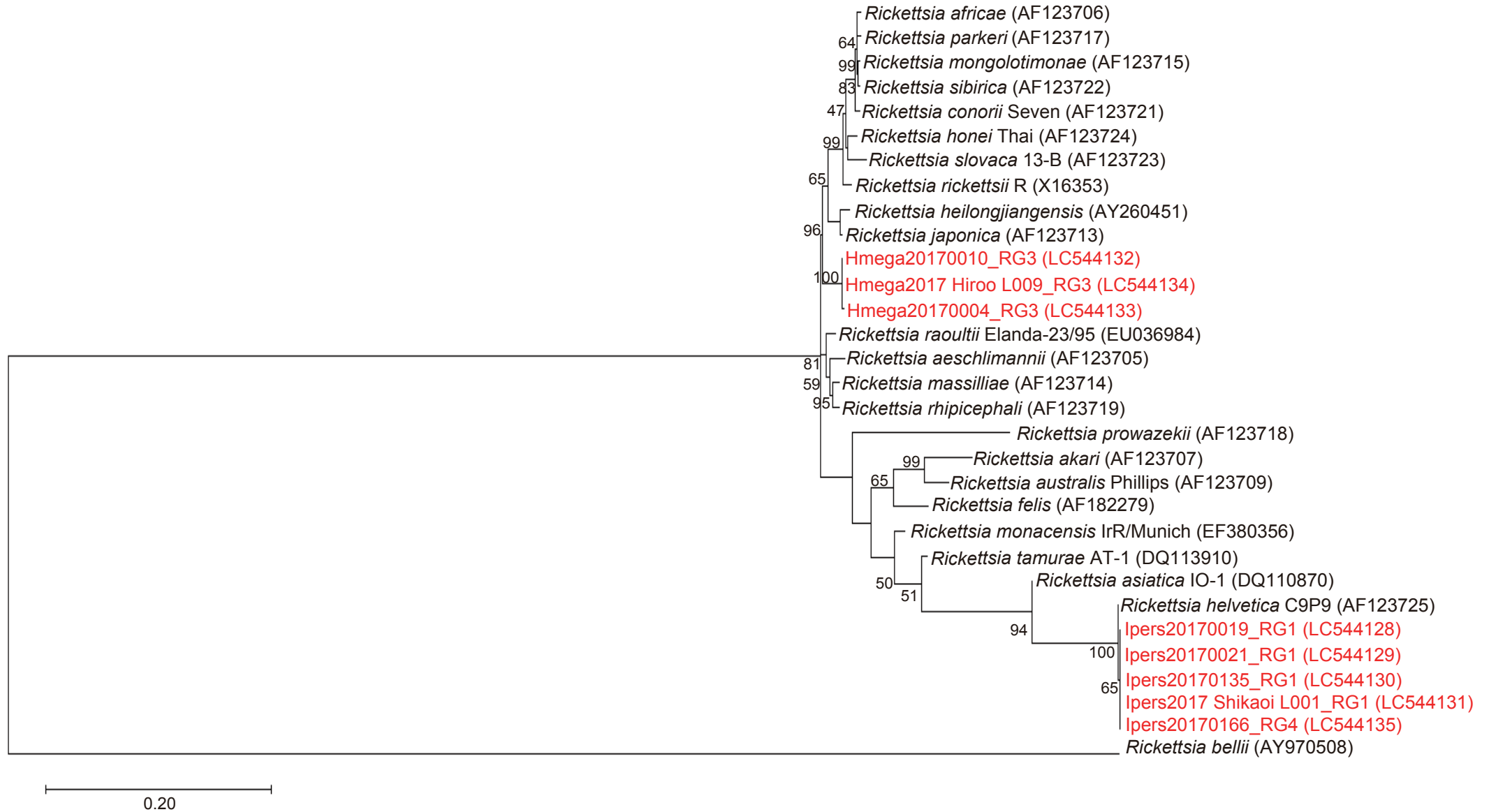

Supplement: Multimedia component 2 — Supplementary Figure S2. Full size image of Fig. 5. [file mmc2.pdf]
