# Supplementary material for: Molecular detection of Borrelia burgdorferi (sensu lato) and Rickettsia spp. in hard ticks distributed in Tokachi District, eastern Hokkaido, Japan
Source: Curr Res Parasitol Vector Borne Dis. 2021 Nov 11;1:100059. doi: 10.1016/j.crpvbd.2021.100059 (PMC8906132; doi:10.1016/j.crpvbd.2021.100059)
Supplement: Multimedia component 3 — Supplementary Table S1. Tick species distribution and prevalence of Borrelia burgdorferi (s.l.) and Rickettsia spp. among questing ticks collected in Tokachi District, eastern Hokkaido, Japan. [file mmc3.pdf]

Supplementary Table S1. Tick species distribution and prevalence of *Borrelia burgdorferi* (*s.l.*) and *Rickettsia* spp. among questing ticks collected in Tokachi district, eastern Hokkaido, Japan.

Month	Province	Species	Stage[a]	Total No.[b]	Tested[c]	*Borrelia burgdorferi* (*s.l.*) (%)			*Rickettsia* spp. (%)		
					(No. pool)	*B. j.*	*B. g./B. b.*	*B. a.*	*R. h.*	"*Ca.* R. t."	"*Ca.* R. p."
May	Memuro	*I. ovatus*	Male	84	78	1 (1.3)	0 (0)	0 (0)	0 (0)	0 (0)	0 (0)
			Female	123	114	32 (28)	0 (0)	0 (0)	0 (0)	0 (0)	0 (0)
		I. persulcatus	Male	51	49	0 (0)	9 (18)	1 (2)	8 (16)	6 (12)	0 (0)
			Female	77	66	0 (0)	20 (30)	2 (3)	28 (42)	12 (18)	0 (0)
			Nymph	7	6	0 (0)	0 (0)	0 (0)	2 (33)	0 (0)	0 (0)
		H. japonica	Male	1	1	0 (0)	0 (0)	0 (0)	0 (0)	0 (0)	0 (0)
			Female	1	1	0 (0)	0 (0)	0 (0)	0 (0)	0 (0)	0 (0)
			Nymph	2	2	0 (0)	0 (0)	0 (0)	0 (0)	0 (0)	0 (0)
Jun	Shikaoi	*I. ovatus*	Male	34	16	0 (0)	0 (0)	0 (0)	0 (0)	0 (0)	0 (0)
			Female	29	0	–	–	–	–	–	–
		I. persulcatus	Male	22	11	0 (0)	1 (9)	0 (0)	0 (0)	4 (36)	0 (0)
			Female	12	6	0 (0)	2 (33)	0 (0)	1 (17)	1 (17)	0 (0)
			Nymph	14	10	0 (0)	2 (20)	0 (0)	1 (10)	0 (0)	0 (0)
			Larva	34	34 (4)	0 (0)	0 (0)	0 (0)	4 (100)	0 (0)	0 (0)
		H. japonica	Male	4	2	0 (0)	0 (0)	0 (0)	0 (0)	0 (0)	0 (0)
			Female	8	0	–	–	–	–	–	–
			Nymph	8	3	0 (0)	0 (0)	0 (0)	0 (0)	0 (0)	0 (0)
			Larva	2	1	0 (0)	0 (0)	0 (0)	0 (0)	0 (0)	0 (0)
Jul	Urahoro	*I. ovatus*	Male	21	21	6 (29)	0 (0)	0 (0)	0 (0)	0 (0)	0 (0)
			Female	38	38	17 (45)	0 (0)	0 (0)	0 (0)	0 (0)	0 (0)
		I. persulcatus	Male	10	10	0 (0)	1 (10)	0 (0)	1 (10)	1 (10)	0 (0)
			Female	4	4	0 (0)	2 (50)	0 (0)	1 (25)	1 (25)	0 (0)
			Nymph	8	2	0 (0)	0 (0)	0 (0)	0 (0)	1 (50)	0 (0)
		H. megaspinosa	Nymph	2	1	0 (0)	0 (0)	0 (0)	0 (0)	0 (0)	0 (0)
			Larva	165	90 (9)	0 (0)	0 (0)	0 (0)	0 (0)	0 (0)	0 (0)
Aug	Shimizu	*I. ovatus*	Male	5	4	0 (0)	0 (0)	0 (0)	0 (0)	0 (0)	0 (0)
			Female	11	10	4 (40)	0 (0)	0 (0)	0 (0)	0 (0)	0 (0)
		I. persulcatus	Female	1	1	0 (0)	0 (0)	0 (0)	0 (0)	0 (0)	0 (0)
		H. japonica	Nymph	1	1	0 (0)	0 (0)	0 (0)	0 (0)	0 (0)	0 (0)
		H. megaspinosa	Nymph	2	2	0 (0)	0 (0)	0 (0)	0 (0)	0 (0)	0 (0)
Sep	Hiroo	*I. persulcatus*	Larva	17	2 (1)	0 (0)	0 (0)	0 (0)	0 (0)	1 (100)	0 (0)
		H. megaspinosa	Male	13	13	0 (0)	0 (0)	0 (0)	0 (0)	0 (0)	2 (15)
			Female	9	4	0 (0)	0 (0)	0 (0)	0 (0)	0 (0)	1 (25)
			Nymph	7	3	0 (0)	0 (0)	0 (0)	0 (0)	0 (0)	0 (0)
			Larva	437	437 (44)	0 (0)	0 (0)	0 (0)	1 (2.3)	0 (0)	2 (4.5)
	Taiki	*I. ovatus*	Male	4	4	1 (25)	0 (0)	0 (0)	0 (0)	0 (0)	0 (0)
			Female	6	6	2 (33)	0 (0)	0 (0)	0 (0)	0 (0)	0 (0)
		I. persulcatus	Male	2	2	0 (0)	0 (0)	0 (0)	1 (50)	0 (0)	0 (0)
			Nymph	2	2	0 (0)	0 (0)	0 (0)	1 (50)	0 (0)	0 (0)
			Larva	16	16 (2)	0 (0)	0 (0)	0 (0)	0 (0)	1 (50)	0 (0)
		H. megaspinosa	Male	14	13	0 (0)	0 (0)	0 (0)	0 (0)	0 (0)	1 (7.7)
			Female	5	4	0 (0)	0 (0)	0 (0)	0 (0)	0 (0)	1 (33)
			Nymph	11	4	0 (0)	0 (0)	0 (0)	0 (0)	0 (0)	0 (0)
			Larva	42	30 (3)	0 (0)	0 (0)	0 (0)	0 (0)	0 (0)	0 (0)

Supplementary Table S1. Tick species distribution and prevalence of *Borrelia burgdorferi* (*s.l.*) and *Rickettsia* spp. among questing ticks collected in Tokachi district, eastern Hokkaido, Japan. (continued)

Month	Province	Species	Stage[a]	Total No.[b]	Tested[c]	*Borrelia burgdorferi* (*s.l.*) (%)			*Rickettsia* spp. (%)		
					(No. pool)	*B. j.*	*B. g./B. b.*	*B. a.*	*R. h.*	"*Ca.* R. t."	"*Ca.* R. p."
Oct	Shikaoi	*I. persulcatus*	Male	1	0	–	–	–	–	–	–
			Nymph	1	0	–	–	–	–	–	–
		H. megaspinosa	Male	1	0	–	–	–	–	–	–
			Nymph	3	0	–	–	–	–	–	–
		H. japonica	Male	1	0	–	–	–	–	–	–
			Female	1	0	–	–	–	–	–	–
			Nymph	2	0	–	–	–	–	–	–
Nov	Ashoro	*I. ovatus*	Female	3	3	1 (33)	0 (0)	0 (0)	0 (0)	0 (0)	0 (0)
			Larva	6	1	0 (0)	0 (0)	0 (0)	0 (0)	0 (0)	0 (0)
		I. persulcatus	Nymph	2	0	–	–	–	–	–	–
			Larva	15	9 (2)	0 (0)	0 (0)	0 (0)	1 (50)	1 (50)	0 (0)
		H. japonica	Male	3	3	0 (0)	0 (0)	0 (0)	0 (0)	0 (0)	0 (0)
			Nymph	109	4	0 (0)	0 (0)	0 (0)	0 (0)	0 (0)	0 (0)
		H. megaspinosa	Nymph	10	1	0 (0)	0 (0)	0 (0)	0 (0)	0 (0)	0 (0)
			Larva	39	10 (2)	0 (0)	0 (0)	0 (0)	0 (0)	0 (0)	0 (0)

[a]Developmental stages of ticks collected.

[b]Total number of ticks collected.

[c]Number of ticks tested for microorganism detection.

Abbreviations: *B. a.*, *Borrelia afzelii*; *B. b.*, *Borrelia bavariensis*; *B. g.*, *Borrelia garinii*; *B. j.*, *Borrelia japonica*; "*Ca*. R. p.", "*Candidatus* Rickettsia principis"; "*Ca*. R. t.", "*Candidatus* Rickettsia tarasevichiae"; *R. h.*, *Rickettsia helvetica*; 0 (0), the tick samples tested were all negative for the microorganism. Hyphens indicate that no tick sample was tested.
